# Charting a path to health for all at net-zero emissions

**DOI:** 10.1016/S0140-6736(25)00813-X

**Published:** 2025-04-25

**Authors:** Judith Rodin, Jennifer R Bratburd, Noah Duff, Jonathan A Patz, Howard Frumkin, Catherine E Woteki, Sarah Whitmee, Michele Toplitz, Victor J Dzau, Andy Haines

**Affiliations:** University of Pennsylvania, Philadelphia, PA, USA; University of Wisconsin-Madison, Madison, WI, USA; National Academy of Medicine, Washington, DC 20001, USA; University of Wisconsin-Madison, Madison, WI, USA; University of Washington, Seattle, WA, USA; Iowa State University, Ames, IA, USA; London School of Hygiene & Tropical Medicine, London, UK; National Academy of Medicine, Washington, DC 20001, USA; London School of Hygiene & Tropical Medicine, London, UK

Climate change is the defining health challenge of the 21st century, with record-breaking temperatures and extreme weather events already exacting an unprecedented toll on human health and wellbeing. Scientific consensus is clear: rapid and deep reductions in greenhouse gas (GHG) emissions by 2050 are needed not only to reduce the risks of exceeding climate tipping points beyond which irreversible damage occurs to natural systems, but also to safeguard human health, wellbeing, and equity.^1,2^ Despite growing awareness of the climate–health nexus, climate interventions often fail to consider opportunities to maximise co-benefits.

Yet, health fundamentally depends on determinants that extend far beyond biomedical interventions—social, economic, and environmental factors shaped by decisions across energy, agriculture, heavy industry, transportation, and building sectors. These sectors are responsible for the overwhelming majority of global GHG emissions and hold the greatest mitigation potential to achieve 2035 emissions targets,^3^ underscoring the need for an integrated systems approach that explicitly accounts for the public health benefits of climate action. Unlike abstract emissions targets or distant ecological concerns, tangible health outcomes from climate mitigation offer immediate, compelling, and widely shared benefits while simultaneously reducing the health risks of climate change.^4^

For example, air pollution linked to burning of fossil fuels contributes to approximately 5 million premature deaths annually and costs the global economy an estimated $8·1 trillion in premature mortality, lost productivity, and higher health-care expenditures.^5,6^ Resource-intensive food systems—driven by high consumption of GHG-intensive red meat and processed foods—are major contributors to GHG emissions, chronic disease, water pollution, land degradation, and biodiversity loss, imposing annual economic costs exceeding US$8 trillion.^7^ Addressing these health and economic impacts in tandem can yield immediate and long-term benefits: transitioning to clean energy mitigates both emissions and pollution-related illness; shifting urban infrastructure away from car-centric design towards active transportation (eg, walking, cycling, and public transit) fosters physical activity while reducing fossil fuel use; and shifting towards plant-based diets enhances nutrition and sustainability.^4^

These economic impacts underscore that climate-induced health costs directly and indirectly affect productivity, labour markets, health-care expenditures, insurance markets, and economic stability. Consequently, businesses, finance institutions, governments, and other non-health sectors increasingly recognise health as intrinsically linked to their operational sustainability, profitability, and resilience.^8^ Articulating these shared economic interests and climate–health co-benefits can forge broader coalitions across divergent economic and political constituencies, thereby accelerating integrated climate–health action.^9^

Fully realising such co-benefits, however, requires more than piecemeal, sector-specific measures. Achieving health for all at net-zero emissions demands a comprehensive systems-level approach encompassing environmental, social, economic, and political dimensions. Narrowly framed climate policies that overlook structural economic inequalities, political inertia, societal inequities, and intergenerational injustices quickly lose effectiveness, durability, and equity by failing to account for the complex interactions among these interconnected systems.^10^ Truly transformative climate–health strategies must incorporate broader systemic determinants—policies, fiscal incentives, governance frameworks, societal norms, power dynamics, and economic structures. For example, relying solely on traditional metrics such as gross domestic product obscures long-term wellbeing by prioritising near-term economic gains over health, equity, and ecological sustainability.^11^ Realising this shift demands rethinking incentives, regulations, urban planning, food systems, technologies, and economic models that explicitly prioritise holistic human health and wellbeing outcomes as well as actions to stabilise the climate and other crucial Earth systems on which our future depends.^12^

**Figure:**
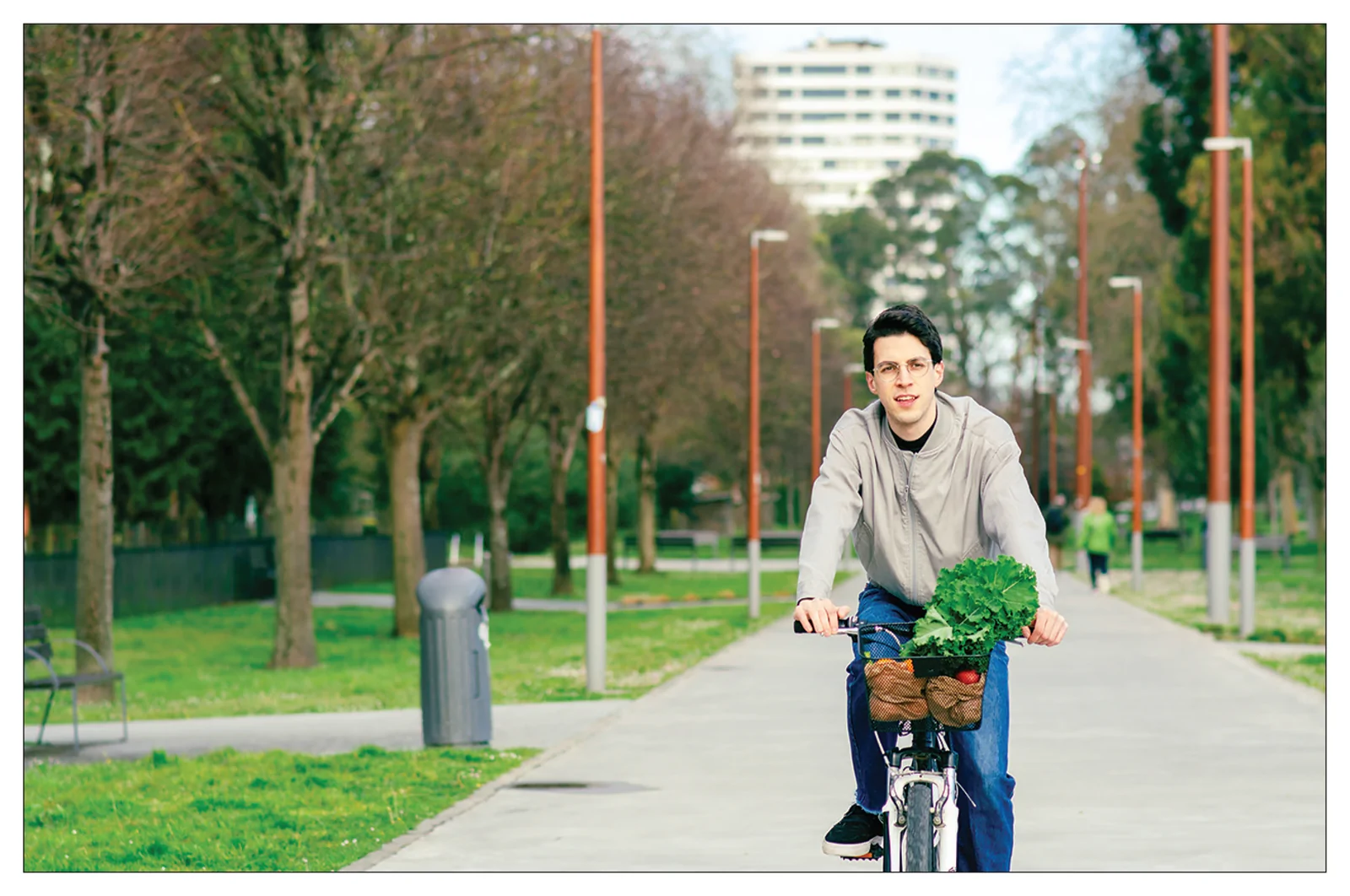


Recognising both the urgency and the opportunity, the US National Academy of Medicine (NAM) is launching a Commission to develop the Roadmap for Transformative Action to Achieve Health for All at Net-Zero Emissions (the Roadmap),^13^ a global initiative designed to position health explicitly at the heart of climate responses. The Roadmap initiative aims to systematically identify high-impact strategies and actionable pathways capable of simultaneously reducing emissions, delivering health gains, promoting equity, and fostering economic resilience and societal wellbeing.

NAM has convened a diverse international Commission representing multiple geographies, sectors, and areas of expertise. The Commission’s approach is purposely cross-sectoral and systems-oriented, recognising the interconnectedness of environmental, social, economic, and political systems in driving sustainable transformations. Throughout 2025, the Commission will assemble rigorous evidence syntheses; conduct policy dialogues, consultations, and expert-led workshops to explore promising strategies; identify key implementation barriers; and understand enabling conditions required for successful action. Insights gathered during these activities will directly inform the final Roadmap report (to be published in 2026), which will detail strategies, priorities for action, implementation pathways, and critical leverage points for systemic transformation.

This process will be grounded in an understanding of socioeconomic and historical context. Low-income and middle-income countries (LMICs), which bear minimal historical responsibility for emissions yet experience disproportionate climate–health impacts,^14^ represent some of the fastest-growing populations and economies.^15^ Targeted investments in sustainable infrastructure and resilient communities can leapfrog carbon-intensive development pathways—accelerating progress towards improved health outcomes, equitable economic growth, and significant reductions in global emissions.^16^ The Roadmap will prioritise learning from and identifying equitable solutions for LMICs, engaging international actors and financing institutions to advance transformative climate–health investments in these contexts.

By explicitly harnessing the motivational power of health—linking it systematically to economic sufficiency, wellbeing, environmental sustainability, societal cohesion, and political resilience—the Roadmap seeks to galvanise broad-based global action towards achieving health for all at net-zero emissions. NAM invites diverse constituencies—including policy makers, industry leaders, researchers, and civil society worldwide—to actively contribute perspectives, evidence, and solutions to inform this global effort.

Charting a successful path towards health for all at net-zero emissions necessitates moving beyond incremental progress and sectoral silos by adopting a holistic, integrated systems approach. The NAM’s forthcoming Roadmap provides a unique opportunity to operationalise this transformative vision, offering actionable guidance, proven strategies, and integrated system-level priorities for decision makers worldwide. By centring health within comprehensive climate strategies—explicitly addressing environmental, economic, social, and political determinants—we aim to catalyse sustained, equitable, and meaningful climate action, protecting human health and fostering resilience and prosperity for all.
